# NGF and TERT Co-Transfected BMSCs Improve the Restoration of Cognitive Impairment in Vascular Dementia Rats

**DOI:** 10.1371/journal.pone.0098774

**Published:** 2014-06-02

**Authors:** Fei Wang, Guangming Chang, Xin Geng

**Affiliations:** 1 Department of Neurology, General Hospital, Tianjin Medical University, Tianjin, China,; 2 Department of clinical laboratory, General Hospital, Tianjin Medical University, Tianjin, China; 3 Department of Biochemistry and Molecular Biology, Tianjin Medical University, Tianjin, China; 4 Tianjin Key Laboratory of Cellular and Molecular Immunology, Tianjin, China; 5 Key Laboratory of Educational Ministry of China, Tianjin, China; University of Nebraska Medical Center, United States of America

## Abstract

Vascular dementia (VaD) is a mental disorder caused by brain damage due to cerebrovascular disease, and incidence of VaD is rising. To date, there is no known effective cure for VaD, so effort in developing an effective treatment for VaD is of great importance. The differentiation plasticity of BMSCs, in conjunction with its weak immunogenicity, makes manipulated BMSCs an attractive strategy for disease treatment. However, BMSCs often display disabled differentiation, premature aging, and unstable proliferation, reducing their neuroprotective function. These problems may be caused by the lack of telomerase activity in BMSCs. Our results show that NGF-TERT co-transfected BMSCs have a better therapeutic effect than BMSCs lacking NGF and TERT expression, demonstrated by significant improvements in learning and memory in VaD rats. The underlying mechanism might be increased expression of NGF, TrkA and SYN in the hippocampal CA1 area, which has potential implication in advancing therapeutics for VaD.

## Introduction

Vascular dementia (VaD) is a mental disorder caused by brain damage due to cerebrovascular disease, and incidence of VaD is rising. In certain Asian countries, VaD is the primary cause of senile dementia. Treatments for VaD are currently under investigation [1]–[3].

Cell replacement therapy is a treatment involving cell supplementation to repair and restore the function of impaired tissues. Stem cell therapy is one method of cell therapy [4], [5]. Bone marrow mesenchymal stem cells (BMSCs) and hematopoietic stem cells are two stem cell populations within the bone marrow. Under certain induction conditions, BMSCs can differentiate into bone, fat, skeletal muscle, astrocytes, or neuronal cells. BMSCs are easily isolated and cultured, proliferate quickly, and have weak immunogenicity, making them ideal seed cells for tissue engineering [6]. Studies have reported that transplanted BMSCs can promote angiogenesis and remodelling of ischemic rat brain tissue. However, BMSCs often display disabled differentiation, premature aging, and unstable proliferation, reducing their neuroprotective function. These problems may be caused by the lack of telomerase activity in BMSCs [7]–[8]. Telomerase reverse transcriptase (TERT), a key catalytic subunit of telomerase, catalyses TTAGGG repeats at the end of a chromosome to maintain telomere length, which is critical for maintaining reproductive activity in cells. TERT is considered for use in increasing the telomerase activity in BMSCs because the TERT expression and telomerase activity are negligible in untransfected BMSCs. Nerve growth factor (NGF) can promote and maintain the growth and survival of endogenous neural cells, differentiate BMSCs into neuron-like cells and play a neuroprotective effect on brain tissue [9].

Nerve growth factor (NGF) and brain-derived neurotrophic factor (BDNF) belong to the family of neurotrophins characterized by the ability to regulate diverse neuronal responses. NGF can enhance the number of synapses by improving the survival of discrete neuronal subpopulations. The exogenous neurotrophins (NGF or BDNF) are introduced into hippocampus significantly improve abilities of spatial learning and memory [10]. However, exogenous NGF can not permeate into blood-brain barrier, so its clinical application is limited for lacking of effective methods of delivering NGF into brain tissues. Developing a simple and practical ways of delivering NGF into the brain tissues continuously and safely has presented new significant challenges [11]. The present study is to test whether BMSCs co-transfected with NGF and TERT have a better therapeutic effect, in terms of significantly restoring memory in VaD rats, than do BMSCs lacking NGF and TERT expression. A comprehensive test of this hypothesis was performed using behavioural, morphological, and molecular biological methods.

## Materials and Methods

### Isolation and identification of rat BMSCs

This study was performed in strict accordance with the recommendations in the guidelines issued by the National Institutes of Health for care of laboratory animals. All experimental protocols were approved by the Committee on the Ethics of Animal Experiments of Tianjin Medical University. All surgery was performed under sodium pentobarbital anaesthesia, and all efforts were made to minimise suffering. Rats were purchased from the Chinese Academy of Military Medical Sciences. Wistar rats, weighing approximately 80 g, were killed under sterile conditions, and then the femoral and tibial sides were isolated, and the ends of long bones were cut. The bone marrow cavity was washed with L-DMEM containing 10% FBS, and suspended cells were collected. The cells were transferred to a 10-cm culture dish and incubated in an incubator at 37°C and 5% CO2. Half of the medium was replaced after three days, and all medium was replaced every 4 days after that. About two weeks later, when the cells reached nearly 90% confluence, the cells were digested with 0.25% trypsin and passaged at a 1∶3 dilution. After five generations, digested cells were collected and washed with PBS. Cells were incubated with FITC-labelled antibodies against CD29, CD31, CD34, CD44, CD45 and CD90. Cells were resuspended in 1% paraformaldehyde prior to flow cytometric analysis.

### Neuronal Induction and differentiation

BMSCs, after 10 weeks in culture, were seeded into six-well plates and pre-induced for 24 h with 20% FBS and 10 ng/mL bFGF in L-DMEM when the cells reached 70% confluence. Then, cells were washed 3 times with PBS and induced for 5 hours with 200 mmol/L BHA and 2% DMSO in L-DMEM.

### BMSCs transfected with TERT-pLXSN

The TERT and pLXSN (a retroviral vector) construct was generated as previously described [12]. After BMSCs were identified, they were seeded into six-well plates and separated into 3 groups, untransfected BMSCs (as a negative control), BMSCs expressing an empty vector, and TERT-transfected BMSCs. When cell confluence reached 90%, 4 µg pLXSN, 10 µl Lipofectamine2000, and 2.5 ml L-DMEM were added to every well. After 6 hours of culture, medium was replaced and the cells were passaged after 24 hr at a 1∶10 dilution. After 48 hours, 200 µg/mL G418 was used for screening. After 10 days, the concentration of G418 was decreased to 50 µg/mL in the control group. TERT mRNA expression levels were analysed by real-time RT-PCR. TERT primer sequences used for real-time RT-PCR are shown in Table 1. Telomerase activity was detected by telomere repeat amplification protocol (TRAP) two months post-transfection.

**Table 1:** Primers in real-time RT-PCR analysis.

Gene Name	Primer Sequences	Product length
NGF	sense: AAT CAA CTC CTG CTT GGC	349 bp
	antisense: GTA TTT AGC CCC TCC TCC	
TrkA	sense: CAT GAC ACT GGG TGG CAG TT	149 bp
	antisense: TCC CCT AGC TCC CAC TTG AGA	
SYN	sense: CCC TAC ATT CAC CCA CTT CTC C	363 bp
	antisense: TTA TCT CCT CTC TGC CCG TTT C	
TERT	sense: 5′-TCCGCACGTT GGTTGCCCAG-3′	203 bp
	antisense: 5′-CCTCTCACCGCGCTCGCA AA-3′	
β-actin	sense: GAG ACC TAC AAG ACC CCA GCC	445 bp
	antisense: TCG GCG CAT CGG TAC CGC TCA	

### MTT assay for detecting cell proliferation

Viable cells can metabolise Thiazolyl Blue Tetrazolium Bromide (MTT) to produce formazan (FM) in the cell, which can be dissolved in dimethylsulphoxide (DMSO) to produce a blue colour. Blue intensity reflects the extent of cell survival and proliferation. After culturing cells for 1 month or 2 months, untransfected, empty-vector-transfected, and TERT-transfected BMSCs were digested with 0.05% trypsin for dispersal into single-cell suspension and seeding into 96-well plates. Each well was seeded with 4000 cells in 200 µl, with 5 individual timepoints performed for each cell type. The absorbance value of each well (OD value) was detected at 570 nm from day 1 to day 5, using a Model 680 microplate reader (BIO-RAD Company). OD values over time were used to construct a growth curve.

### Soft agar colony formation assay

Colony formation assay in soft agar was used to detect the tumorigenicity of BMSCs in vitro. DMEM (3 ml) containing 0.9% agarose was added to the bottom of each well of a six-well plate, and the plate was placed in the incubator to solidify overnight. Logarithmically growing cells after 30 generations were digested and counted. Low-melting-point agar (0.36%) containing 10% FBS-DMEM was prepared and mixed with 2×10^4^ cells suspended in 2 ml DMEM. The 6-well plates were incubated overnight. Then, 200 µl DMEM containing 10% FBS medium was added to each well. The numbers of cloned cells were counted under an inverted microscope after 4–6 weeks, and C6 glioma cells were used as a positive control. C6 glioma cell line was obtained from Institute of Neurology, General Hospital, Tianjin Medical University.

### TERT-BMSCs transfected with NGF

The recombinant NGF adenoviral vector was constructed as previously described [13]. TERT-BMSCs were digested with 0.25% trypsin and seeded to approximately 1×10^6^ cells per well. Ad-NGF (75 µl) was added to the culture dish at a multiplicity of infection (MOI) of 100 pfu/cell. After 2 hours of incubation, 20% FBS DMEM medium was added, and cells were then incubated for 48 hours. NGF expression was detected by immunofluorescence staining.

### VaD animal models and BMSCs transplantation

Forty-eight healthy male Wistar rats, weighing 260–300 g, were randomly and equally divided into four groups: control group, VaD group, untransfected BMSCs transplanted VaD group (untransfected BMSCs transplanted to VaD rats) and NGF-TERT-BMSCs transplanted VaD group (NGF-TERT co-transfected BMSCs transplanted to VaD rats). Four to six rats per cage, were kept with the temperature maintained at 25±1°C, given water, adequately fed, and exposed to 12 hours light, 12 hours dark. All conditions conform to the laboratory animal care guidelines issued by National Institutes of Health (NIH), USA.

Two-vessel occlusion (2VO), which is the most popular method for generating VaD rat models, was used to produce our VaD rat model. The surviving rats were tested in the Morris water maze 2 days after the operation. Using the reference value of the full-time average escape latency of the rats in the control group, we calculated the following proportion: one minus the reference value divided by the full average escape latency of the combined experimental groups (including the VaD group, the untransfected BMSCs transplanted VaD group and the NGF-TERT–BMSCs transplanted VaD group). If the proportion >20%, the rat is regarded as having VaD, with >20≤30% considered mild dementia, >30≤40% moderate dementia, and >40% severe dementia [14].

Untransfected and NGF-TERT co-transfected BMSCs were injected to the rats in untransfected BMSCs transplanted VaD group and NGF-TERT co-transfected BMSCs transplanted VaD group, individually, via tail vein at a concentration of 4×10^6^ cells/ml.

### Water Maze Test of Spatial Learning and Memory

The Morris water maze test was performed as previously described [15]. Morris water maze test methods include the positioning navigation test and the spatial exploration test. On the first day of the positioning navigation test, rats were allowed to swim freely for 2 min to become familiar with the environment, without a platform. The rats' swimming speed was observed and the rats with poor swimming ability were removed. On days 2-4, the period starting when the rats were put into the water and ending when they found and climbed onto the platform was called the full average escape latency. The distance from the rats' entry into the water to the end location was called the swimming distance. The test was divided into two sessions per day, at intervals of 8 hours. The rat was considered to have found the platform if it remained there for 2 s. If the rat did not find the platform within 120 s, it was led to the platform and kept there for 20 s to become familiar with the environment and the platform position, and the escape latency was recorded as 120 s. On the fifth day, the spatial exploration test was performed. The platform was removed, and the trajectory was recorded for 2 min beginning with the rat's entry into the water from the first quadrant. The swimming time in the original platform quadrant and the percentage of the swimming distance in the original platform quadrant were calculated.

### RNA extraction and Quantitative Real-Time RT-PCR

Total RNA was extracted from brain tissues using TRIZOL reagent (Gibco, Invitrogen Corp.) according to the manufacturer's instructions. Super-Script reverse transcriptase (Gibco, Invitrogen Corp.) was used to synthesise cDNA. The mRNA expression levels of the NGF, TrkA (Tyrosine Receptor Kinase A), and SYN (synaptophysin) genes were detected by quantitative real-time RT- PCR, and amplification of β-actin mRNA was performed in each sample as an internal control. The nucleotide sequences of the primers are shown in Table 1. Quantitative real-time PCR was performed using a Light-Cycler PCR system (Roche Diagnostics Ltd.) with protocols provided by the manufacturer. RT-PCR reactions were performed in a total volume of 20 µL, including 2 µl cDNA at a 1∶10 dilution, 0.5 µM primers, and SYBR Green I mix. The amplification protocol consisted of one incubation at 95°C for 10 min followed by 30 cycles at 94°C for 30 s, 58°C for 30 s, and 72°C for 30 s. The fluorescent products were detected at the end of the final extension period. To confirm the specificity of amplification, the PCR products from each primer pair were subjected to melting curve analysis and subsequent sequence analysis. To exclude genomic DNA contamination, the PCR products produced both with and without RT with NGF, TrkA, SYN, and β-actin primers were electrophoresed on 1.5% agarose gels and stained with ethidium bromide. The amounts of NGF, TrkA and SYN mRNA were normalised against mRNA levels of the housekeeping gene β-actin in the corresponding samples.

### Immunohistochemical Analysis

Brain tissues were fixed with 4% paraformaldehyde, dehydrated with a conventional alcohol gradient, paraffin-embedded, and cut into 4-µm-thick consecutive coronal slices, which were mounted on clean slides pre-treated with poly-L-lysine. The slices were baked in a 60°C oven for 2 h for later use. DAB immunohistochemistry was performed to detect the expression of NGF, TrkA and SYN proteins in the hippocampal CA1 area. The results of immunohistochemical staining were observed with the Leica Mias 2000 image analysis system, and the average grey values of the staining product were determined by the image analysis software (Mias99 Version 3.0).

### Western blot analysis

Brain tissues were homogenised in lysis buffer containing Tris-HCl (pH 7.4), protease inhibitor, and 0.5% Triton X-100. Concentrations of proteins were measured using the Bradford method. Samples containing 50 µg/mL of total protein were resuspended in loading buffer [0.1 M of Tris-HCl (pH 6.8), 4% sodium dodecyl sulfate, 0.2 M of DTT, 20% glycerol, and 0.2% bromophenol blue], denatured by boiling for 10 min, and separated by SDS-PAGE. The proteins were transferred to Trans-Blot polyvinylidene fluoride membranes (Applied Biosystems, USA), and membranes were probed separately with anti-NGF polyclonal antibody, anti-TrkA polyclonal antibody, and anti-SYN polyclonal antibody (BD Biosciences Pharmingen, USA), at a dilution of 1∶500 for 3 h. Blots were washed in TBST (50 mM Tris [pH 7.5], 150 mM NaCl, and 0.1% Tween-20) and incubated with secondary antibody for 1 h. Enhanced chemiluminescence (Thermo Fisher Scientific Inc., USA) was performed, followed by exposure to X-ray films. Anti-actin was used for normalisation, and relative amounts of NGF/actin, TrkA/actin, and SYN/actin were calculated.

### Transmission electron microscopy (TEM)

In accordance with Pellegrino's three-dimensional map, the hippocampal CA1 area of the rats in each group (n = 12) were quickly removed, immediately put into 2% glutaraldehyde liquid (containing 3% fresh poly-L-lysine), fixed with 1% osmium tetroxide for 1 h, dehydration-fixed with ethanol and acetone, embedded in Epon 812, and sliced with an LKBNOVA ultra-thin microtome. The slices were double-stained by uranyl acetate and lead citrate, followed by analysis with a JEM-1010 transmission electron microscope.

## Results

### Isolation and identification of BMSCs

A small number of adherent cells were observed at 24–72 h after seeding of BMSCs, and the cells gradually gathered in the subsequent 2 weeks. The cells had a fusiform shape until the fifth generation. By flow cytometry, 98.32%, 99.27%, and 97.92% of the cells were positive for the surface markers CD29, CD44, and CD90, respectively. Furthermore, CD31, CD34, and CD45 antigens were negative. These results showed that the isolation of BMSCs was successful.

### TERT mRNA expression and telomerase activity in TERT-BMSCs

**Figure 1:**
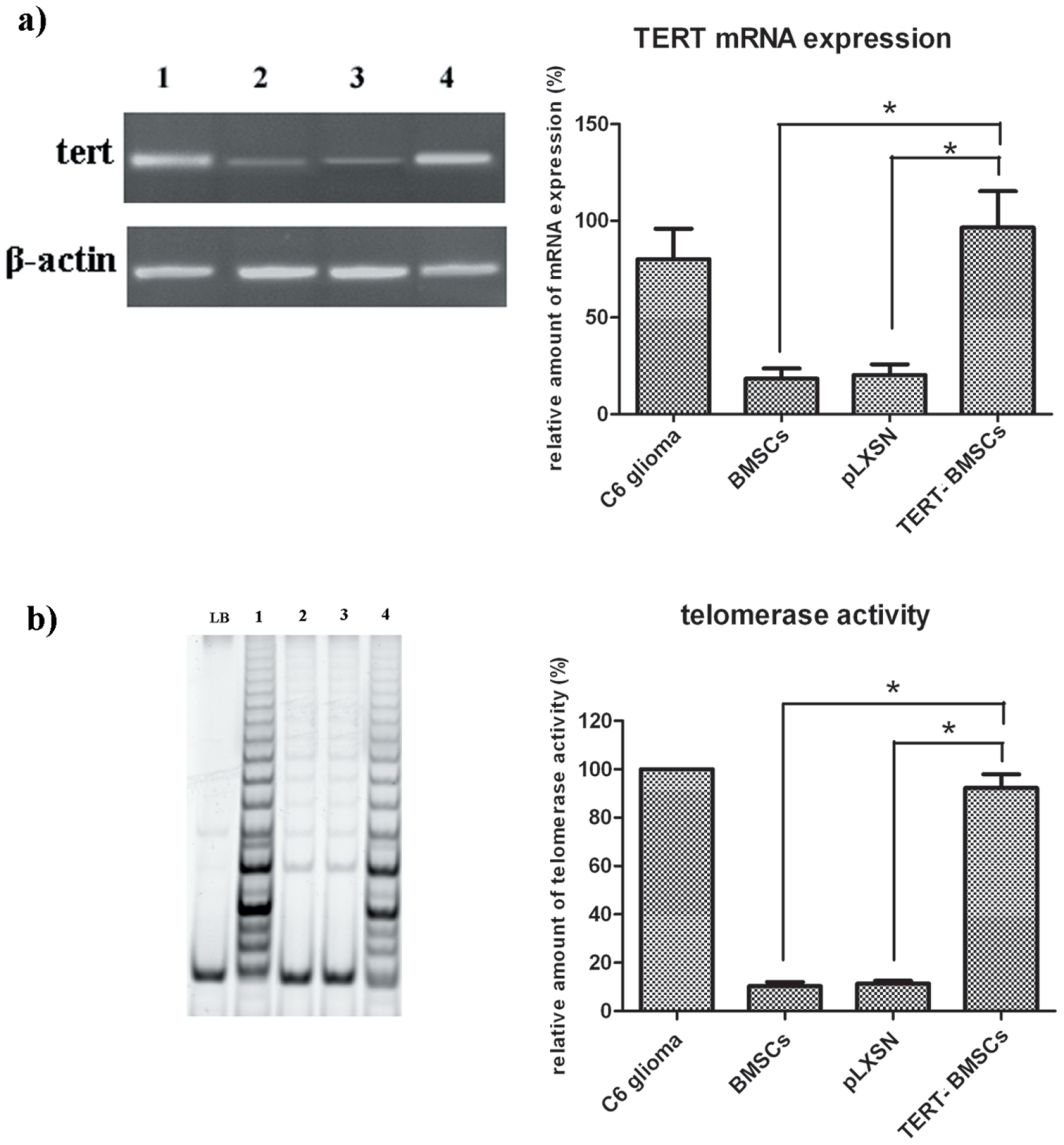
TERT mRNA expression and telomerase activity in TERT-BMSCs. TERT mRNA expression and telomerase activity were significantly increased in TERT-BMSCs compared to untransfected BMSCs (*P<*0.05). a), tert and β-actin mRNA expression by RT-PCR analysis. b), telomerase activity analysis by TRAP. LB, lysis buffer (as a negative control); 1, C6 glioma cell line (as a positive control); 2, untransfected BMSCs; 3, BMSCs transfected with empty pLXSN; 4, TERT-BMSCs (transfected with recombinant TERT-pLXSN).

TERT mRNA expression and telomerase activity were significantly increased in TERT-BMSCs compared to untransfected BMSCs (*P<*0.05,). TERT mRNA expression levels and telomerase activity in the C6 glioma cell line was used as a positive control. (Figure 1a, 1b). These results showed that TERT was successfully transfected into BMSCs and there were more telomerase activity in TERT overexpressed BMSCs.

### Cell proliferation of TERT- BMSCs

**Figure 2:**
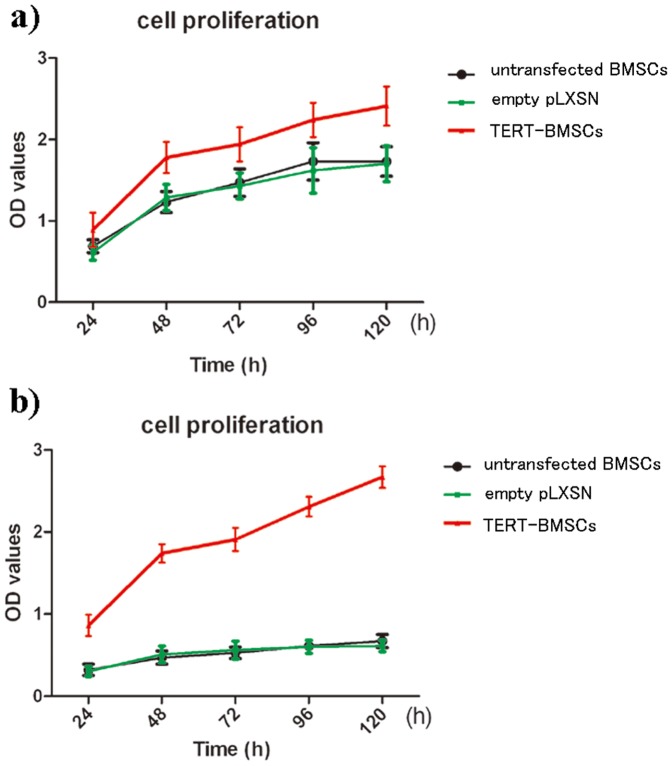
Cell proliferation analysis was performed after cell culture for 1 month (**Figure 2a**) and 2 months (**Figure 2b**). The rate of proliferation in TERT-BMSCs was significantly increased compared to untransfected BMSCs and empty vector-transfected BMSCs following one month of cell culture (*P<*0.05) (Figure 2a). After long-term culture of 2 months, the rate of proliferation decreased in untransfected and empty-vector-transfected BMSCs but remained high in TERT-BMSCs (*P<*0.05) (Figure 2b).

The rate of proliferation in TERT-transfected BMSCs was significantly increased compared to untransfected BMSCs and empty vector-transfected BMSCs following one month of cell culture (*P<*0.05) (Figure 2a). After long-term culture for 2 months, the rate of proliferation decreased in untransfected and empty vector-transfected BMSCs but remained high in TERT-transfected BMSCs (*P<*0.05) (Figure 2b).

### Soft agar colony formation assay

Colony formation was apparent with the C6 glioma positive-control cells, but there was no colony formation in untransfected or TERT-transfected BMSCs. This indicates that C6 glioma cells have a high resistance to low-nutrition medium and therefore a high propensity for colony formation in this assay. However, in vitro, neither untransfected nor TERT -transfected BMSCs had this tumorigenic capacity. Rats transplanted with untransfected or TERT transfected BMSCs did not die showed no tumour formation 24 weeks after transplantation.

### Results of Morris water maze test after treatment with NGF-TERT co-transfected BMSCs

**Figure 3:**
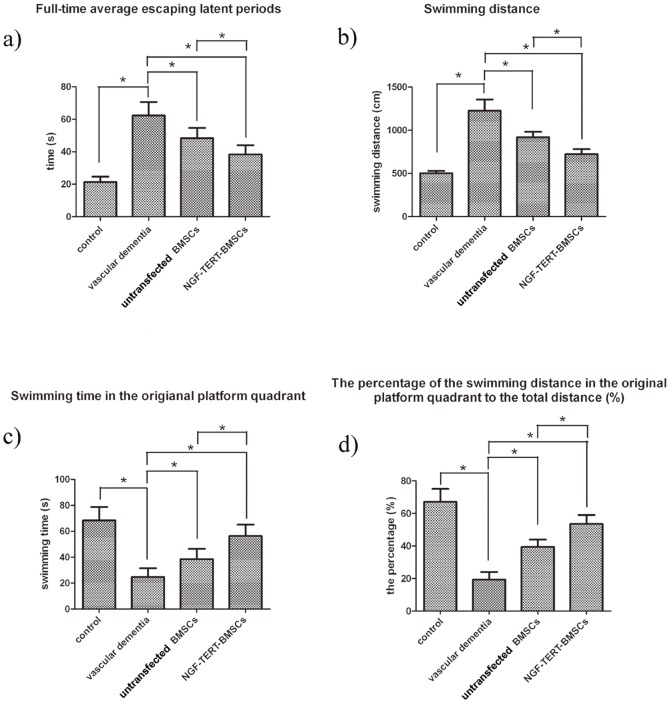
The results of the Morris water maze test in four groups. In the positioning navigation test, the full-time average escape latency period and swimming distance of untransfected and NGF-TERT co-transfected BMSC transplanted VaD groups during three consecutive days were significantly shorter than those of the VaD group (Figure 3a). Escape period and swimming distance of the NGF-TERT co-transfected BMSC transplanted VaD group were significantly shorter than those of the untransfected BMSC transplanted VaD group (Figure 3b). In the space exploration test, the VaD rats treated with untransfected or NGF-TERT co-transfected BMSCs showed significantly longer swimming time in the original platform quadrant and significantly higher percentages of swimming distance in the original platform quadrant than those of the VaD group (Figure 3c). Additionally, the VaD rats treated with NGF-TERT co-transfected BMSCs exhibited significantly longer swimming times and distances in the original quadrant than the rats treated with untransfected BMSCs (Figure 3d) (*P<0.05).

In the positioning navigation test, the full average escape latency and swimming distance of the untransfected and NGF-TERT co-transfected BMSCs transplanted VaD groups during three consecutive days were significantly shorter than those of the VaD group (*P<*0.05). Additionally, the escape latency and swimming distance of the NGF-TERT-BMSC transplanted VaD group were significantly shorter than those of the untransfected BMSC transplanted VaD group (*P<*0.05) (Figure 3a, Figure 3b).

In the space exploration test, the swimming time in the original platform quadrant and the percentage of the swimming distance in the original platform quadrant for the untransfected and NGF-TERT co-transfected BMSC transplanted VaD groups were significantly longer than those of VaD group (*P<*0.05). Additionally, the NGF-TERT co-transfected BMSC transplanted VaD group exhibited significantly longer swimming times and distances in the original quadrant than the untransfected BMSC transplanted VaD group (*P<*0.05) (Figure 3c, Figure 3d).

### Quantitative Real-Time RT-PCR for mRNA expression

**Figure 4:**
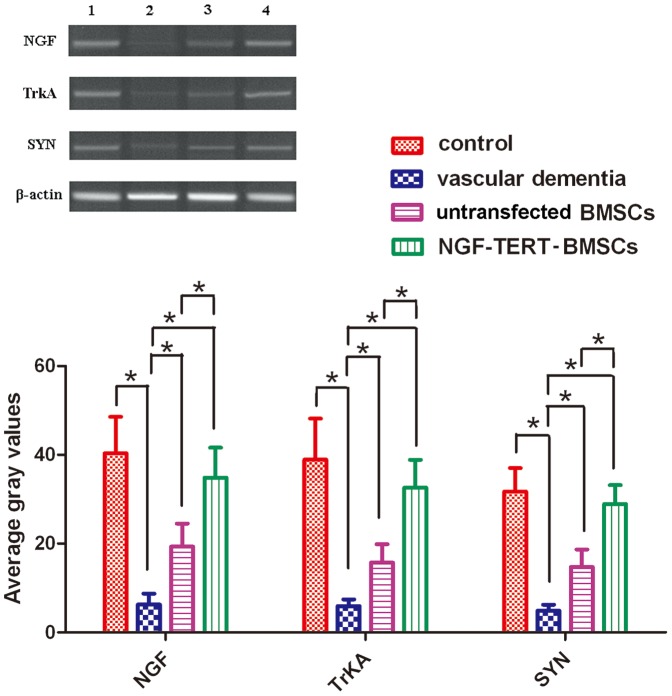
mRNA expression analysis by quantitative real-time RT-PCR. mRNA expression levels of NGF/β-actin, TrkA/β-actin, and SYN/β-actin in the VaD group were significantly lower than those in the control group. Expression levels in untransfected and NGF-TERT co-transfected BMSC transplanted VaD groups were significantly higher than those of the VaD group. Additionally, expression levels in rats treated with NGF-TERT co-transfected BMSCs were significantly higher than those in rats treated with untransfected BMSCs (*, *P<*0.05, n = 12).

mRNA expression levels of NGF/β-actin, TrkA/β-actin, and SYN/β-actin in the VaD model group were significantly lower than those in the control group (*P<*0.05). Expression levels in untransfected and NGF-TERT co-transfected BMSC transplanted VaD groups were significantly higher than those in the VaD group (*P<*0.05). Additionally, expression levels in the NGF-TERT co-transfected BMSC transplanted VaD group were significantly higher than those in the untransfected BMSC transplanted VaD group (*P<*0.05) (Figure 4).

### Western blot analysis for protein expression

**Figure 5:**
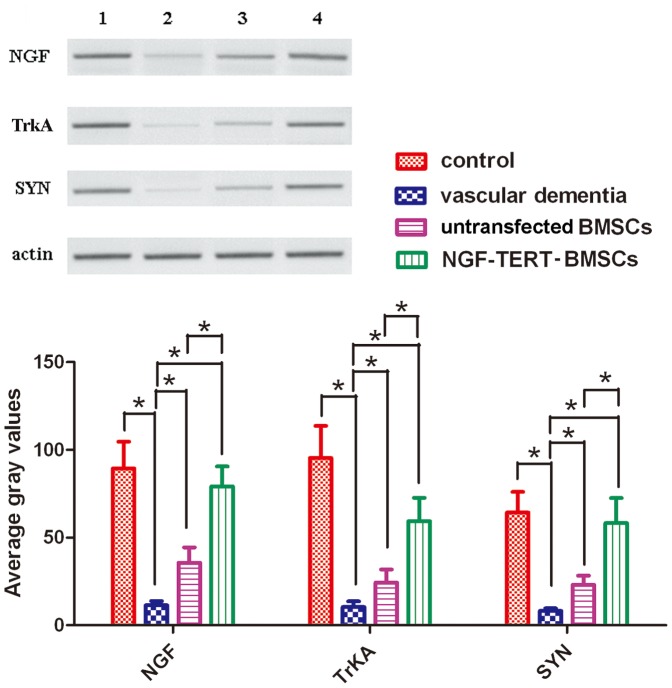
Protein expression analysis by western blotting. After normalisation to actin, protein levels of NGF, TrkA, and SYN in the VaD group were significantly lower than those in the control group. The untransfected and NGF-TERT co-transfected BMSC transplanted VaD groups had significantly higher levels of these proteins than the VaD group. Additionally, rats treated with NGF-TERT co-transfected BMSCs had significantly higher levels of NGF, TrkA, and SYN than rats treated with untransfected BMSCs (*P<0.05, n = 12).

After normalisation to actin, protein levels of NGF, TrkA, and SYN in the VaD model group were significantly lower than those in the control group (*P<*0.05). Untransfected and NGF-TERT co-transfected BMSC transplanted VaD groups had significantly higher levels of these proteins than the VaD group (*P<*0.05). Additionally, the NGF-TERT co-transfected BMSC transplanted VaD group had significantly higher levels of NGF, TrkA, and SYN than the untransfected BMSC transplanted VaD group (*P<*0.05) (Figure 5).

### Immunohistochemical results

**Figure 6:**
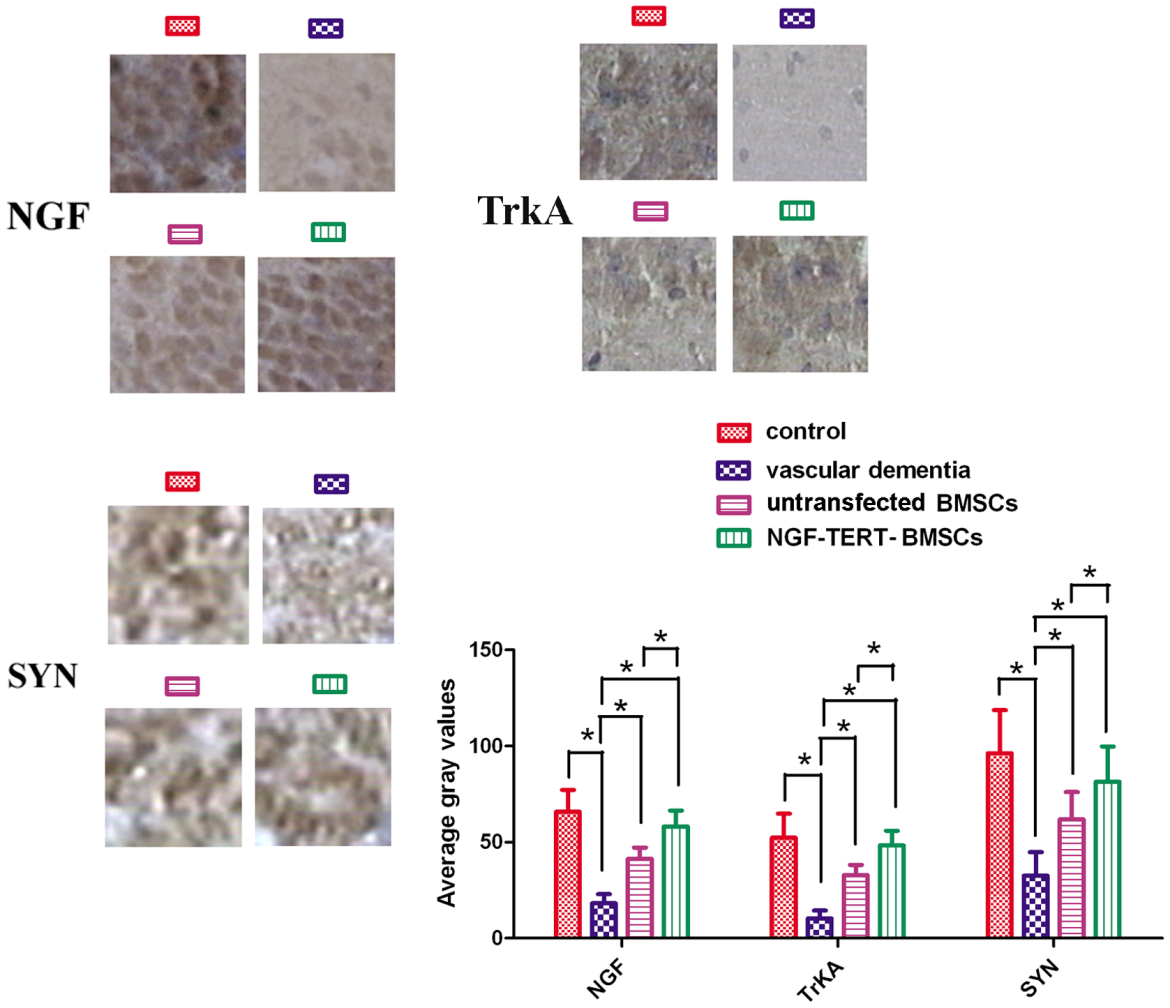
Immunohistochemical analysis of protein expression. NGF-positive and TrkA-positive cells were detected in the hippocampus and cortex, and these proteins were most highly abundant in the cytoplasm of hippocampal cells. SYN protein expression was stronger in the emitting layer of the hippocampus CA1 area than in the molecular layer, and the outline of the cone cells showed minimal SYN. The average grey values of NGF, TrkA, and SYN proteins in the VaD group were significantly lower than those in the control group. The average grey values of untransfected and NGF-TERT co-transfected BMSCs transplanted VaD groups were higher than those of the VaD group. Additionally, grey values of NGF-TERT co-transfected BMSCs transplanted VaD rats were significantly higher than those of the untransfected BMSCs transplanted VaD rats (*P<0.05, n = 12).

NGF-positive and TrkA-positive cells were detected in the hippocampus and cortex, and these proteins were most highly abundant in the cytoplasm of hippocampal cells. SYN protein expression was stronger in the emitting layer of the hippocampus CA1 area than in the molecular layer, and the outline of the cone cells showed minimal SYN. The average grey values for NGF, TrkA, and SYN proteins in the VaD group were significantly lower than those in control group (*P<*0.05). The average grey values for the untransfected and NGF-TERT co-transfected BMSCs transplanted VaD groups were higher than those of the VaD group. Additionally, grey values of the NGF-TERT co-transfected BMSC transplanted VaD group were significantly higher than those of the untransfected BMSC transplanted VaD group (*P<*0.05) (Figure 6).

### TEM for ultrastructure in Hippocampal CA1 area

**Figure 7:**
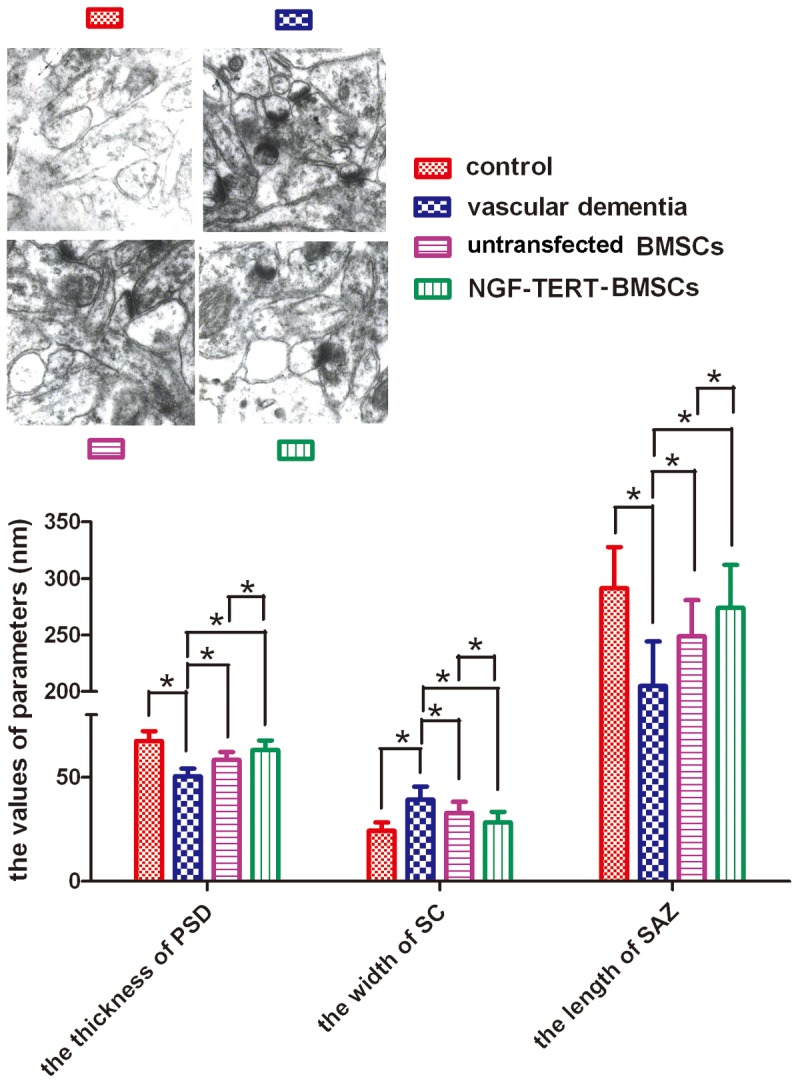
Ultrastructure in the hippocampal CA1 area by transmission electron microscopy (×40,000). The synaptic structural parameters of the hippocampal CA1 area in each group are shown. PSD, postsynaptic density; SC, synaptic cleft; SAZ, synaptic active zone. (*P<0.05, n = 12).

As observed by TEM, the ultra-structure of synapses in the VaD group was disordered and obviously damaged in comparison with the control group. The number of synapses in the hippocampal CA1 area of the VaD group was higher than in controls, but the synaptic volume was significantly decreased, and the synapses were longer or deformed, particularly in the anterior region. Additionally, the number of synaptic vesicles was reduced, and they were aggregated and showed condensed mitochondria. Furthermore, the postsynaptic region was reduced in volume, and the synaptic membrane was thickened and fused, with a disappearance in synaptic space. These defects were improved not only in the untransfected BMSC transplanted VaD group but also in the NGF-TERT co-transfected BMSC transplanted VaD group. In the hippocampal area CA1 of the untransfected and NGF-TERT co-transfected BMSC transplanted VaD groups, synapses were spherical and more regular than in the VaD model group. The presynaptic area was rich in vesicles, which were evenly distributed. The level of mitochondrial aggregation was reduced in the presynaptic area. The synaptic membrane was slightly thickened, the synaptic cleft was more identifiable, and some synaptic structures were improved in the NGF-TERT co-transfected BMSC transplanted VaD group compared with the VaD group and the untransfected BMSC transplanted VaD group. The synaptic ultrastructural parameters of the hippocampal CA1 area in each group are shown in Figure 7.

## Discussion

VaD is a cerebrovascular disease that occurs almost entirely in the elderly. Patients with VaD generally experience a decline in cognitive function [16], [17]. The molecular mechanism of VaD onset is still not clear, and this has hindered recent progress in the development of treatments. There are still no effective treatments for VaD. The main method for the treatment of VaD is to protect the nerve cells in the ischemic penumbra by improving cerebral circulation, oxygen supply and cell metabolism of brain tissues to prevent further infarction. Previous studies suggest that acetylcholinesterase inhibitors, calcium antagonists, and antioxidants are effective in the treatment of VaD [18], [19]. Our previous study reported that repetitive transcranial magnetic stimulation (rTMS) can be beneficial in restorative treatment for VaD. The underlying mechanism may be that rTMS can increase the mRNA and protein expression levels of BDNF, NMDAR1, and SYN in the hippocampal CA1 area [15].

Telomerase is a ribonucleoprotein enzyme and plays a critical role in the replication of chromosome ends in most eukaryotes. It is active in progenitor and cancer cells but is inactive, or has very low activity, in normal somatic cells [20], [21]. TERT, the catalytic component of the telomerase holoenzyme complex, has the main activity of telomere elongation and adds simple sequence repeats to chromosome ends by copying a template sequence within the hTR (RNA component of the enzyme) [22], [23]. Bernardes et al. tested the effects of the telomerase gene therapy in adult and old mice. They treated the adult and old mice with an adeno-associated virus (AAV) expressing mouse TERT, finding beneficial effects on health, including several molecular biomarkers of aging. The telomerase-treated mice, both adult and old, had an increase in median lifespan of 24% and 13%, respectively. Furthermore, telomerase-treated mice did not develop more cancer than the control group [24].

Methods using BMSCs have become the new strategy for treatment of central nervous system diseases. Research using BMSCs is focused on their safety and effectiveness [25], [26]. BMSCs have weak immunogenicity and can differentiate into neurons under inducing conditions in vivo or in vitro. BMSCs may have two primary features in the treatment of dementia. First, BMSCs penetrate the blood-brain barrier and migrate to brain tissue. There, they can differentiate into nerve cells and play roles in neuroprotection. Second, BMSCs increase the secretion of NGF and brain-derived neurotrophic factor (BDNF) and promote the maturation and differentiation of neurons, thereby promoting restoration of nerve damage [27], [28]. Previous studies have confirmed the therapeutic effects of BMSC transplantation in neurological disease. However, the proliferative, differentiative, and homing capacities of BMSCs from the elderly are significantly reduced, especially after several passages and expansion in vitro [29]. Tang et al. [30] reported extended lifespan, enhanced angiogenic capacity, and less tumorigenicity in BMSCs from aged donors when the cells were co-transfected with lentivirus-mediated TERT and VEGF genes. Although there is very less tumorigenicity of TERT-BMSCs, the safety of using TERT overexpressed BMSCs should be considered in the future application. In our present study, we exogenously expressed telomerase in BMSCs utilising a cationic liposome method. Our finding verified TERT overexpression was really making telomerase more active and telomeres longer in vivo. Telomeres length significantly increased in TERT-BMSCs compared to untransfected BMSCs (data were not shown). TERT- BMSCs showed extended lifespan and avoided premature ageing due to the high telomerase activity.

NGF plays important roles in neuronal survival and growth in the brain. Several studies have reported a positive correlation between NGF expression and learning abilities [31], [32]. The roles of NGF in learning and memory are mediated mainly via its activation of the high-affinity receptor TrkA. The conditional TrkA knockout mice displayed selective impairments in cognitive function [33]. The mechanisms by which NGF may improve synaptic plasticity are manifold. NGF can enhance synaptogenesis, resulting in improving the abilities of learning and memory [34]. Furthermore, NGF inhibits apoptosis and can reduce the loss of neurons caused by ischaemia. After NGF binds its specific receptor TrkA, the activation of TrkA induces receptor autophosphorylation, recruitment of adaptor proteins and subsequent activation PI3K or PLCγ, resulting in improving neuronal differentiation and growth and axon formation. The activated PLCγcan also activate MAPK, thus indirectly promoting synapse formation and neuronal differentiation [35]. Birch et al. [36], [37] reported that chronic infusion of NGF could improve learning and memory associated with specific cellular changes in the hippocampus, including synaptogenesis and cell proliferation. The expression levels of NGF and TrkA were increased in NGF-infused rats, and the synaptic vesicle protein synapsin was upregulated. Finally, they observed an increase in cell proliferation in the dentate gyrus of NGF-treated rats.

In the central nervous system, the structural, quantitative and functional states of synapses are closely related to learning and memory outputs [38]–[40]. Damage to synapses occurs during the early stage of VaD,and the extent of the damage is associated with the degree of impaired cognitive function. Several studies reported that the expression and distribution of SYN can indirectly reflect synaptic density [41], [42]. In our present study, the expression level of SYN was significantly higher in rats treated with BMSCs coexpressing NGF and TERT than in other groups, and this correlated with improved performance in the water maze test. After the treatment of VaD rats with untransfected and NGF-TERT co-transfected BMSCs, learning and memory improved significantly. The mRNA and protein levels of NGF, TrkA and SYN in rats treated with either untransfected or NGF-TERT co-transfected BMSCs were higher than those in the VaD rats, and the expression in the NGF-TERT-BMSC transplanted VaD group was significantly higher than that in the untransfected BMSC transplanted VaD group. The synaptic ultrastructure in the hippocampal CA1 area of the NGF-TERT co-transfected BMSC transplanted VaD group was significantly improved compared with those in both the VaD group and the untransfected BMSC transplanted VaD group.

In the present study, we addressed the possibility of transplanting BMSCs co-transfected with NGF and TERT in rats with VaD, as a therapeutic approach to improve cognitive function. To date, there is no known effective cure for VaD. Patients with VaD often present similar symptoms as Alzheimer's disease (AD). Ongoing research in the field includes investigating if medications for AD will also be effective for VaD. However, as VaD and AD harbor different pathological pathways, effort in developing an effective treatment for VaD is of great importance. The differentiation plasticity of BMSCs, in conjunction with its weak immunogenicity, makes manipulated BMSCs an attractive strategy for disease treatment. Based on the results from a combination of water maze and various molecular biology techniques such as real time RT-PCR, western blot, TRAP assays, soft agar and immunohistochemical assays, we concluded that NFG-TERT co-transfected BMSCs improve cognitive ability in VaD rats in the hippocampal CA1 area, which had potential implication in advancing therapeutics for VaD.
